# Musculoskeletal healthcare at a Swiss university hospital chiropractic medicine outpatient clinic in 2019: a health services research study

**DOI:** 10.1186/s12998-022-00417-5

**Published:** 2022-02-11

**Authors:** Léonie Hofstetter, Melanie Häusler, Malin Mühlemann, Luana Nyirö, Daniel Mühlemann, Cesar A. Hincapié

**Affiliations:** 1 Department of Chiropractic Medicine, Faculty of Medicine, Balgrist University Hospital and University of Zurich, Forchstrasse 340, 8008 Zurich, Switzerland; 2 Department of Epidemiology, Biostatistics and Prevention Institute (EBPI), University of Zurich, Zurich, Switzerland

**Keywords:** Musculoskeletal pain, Chiropractic, Outpatient care, Health services research, Outcome assessment, Electronic health records

## Abstract

**Background:**

The Balgrist University Hospital in Zurich, Switzerland, is an academic hospital focused on musculoskeletal disorders. An integrated chiropractic medicine clinic provides chiropractic care to a broad patient population. This health services research study aims to advance understanding of chiropractic healthcare service for quality assurance and healthcare quality improvement.

**Methods:**

We performed an observational clinical cohort study at the Balgrist chiropractic medicine outpatient clinic in 2019. The records of all patients with initial visits or returning initial visits (> 3 months since last visit) and their subsequent visits from January 1, 2019, to December 31, 2019, were used to create the study dataset. Data collected included demographic characteristics, diagnoses, imaging data, conservative treatments, surgeries, and other clinical care data. Descriptive statistics were used to summarize data.

**Results:**

1844 distinct patients (52% female, mean age 48 ± 17 years) were eligible and included in the study. 1742 patients had a single initial visit, 101 had 2 initial visits, and 1 patient had 3 initial visits during the study period (total of 1947 initial visit records). The most common main diagnoses were low back pain (42%; 95% CI 40–46%), neck pain (22%; 20–24%), and thoracic pain (8%; 7–9%). 32% of patients presented with acute (< 4 weeks) symptoms, 11% subacute (4–12 weeks), and 57% chronic (> 12 weeks). Patients had a median of 5 chiropractic visits during their episode of care within a median of 28 days duration. Only 49% (95% CI 47–52%) of patient records had a clinical outcome that was extractable from routine clinical documentation in the hospital information system.

**Conclusion:**

This health services study provides an initial understanding of patient characteristics and healthcare delivered in a Swiss academic hospital chiropractic outpatient setting and areas for improved clinical data quality assurance. A more concerted effort to systematically collect patient reported outcome measures would be a worthwhile healthcare quality improvement initiative.

**Supplementary Information:**

The online version contains supplementary material available at 10.1186/s12998-022-00417-5.

## Background

The increasing prevalence of non-communicable chronic diseases is a major public health challenge worldwide. Musculoskeletal (MSK) conditions are some of the leading causes of global disability, accounting for 16% of the total disability burden [1]. In Switzerland, MSK conditions are one of the six most common non-communicable diseases [2], causing high direct and indirect costs and accounting for 30% of healthcare costs overall [3]. Specifically, the total Swiss economic burden of low back pain (LBP) per year was estimated at 6.6 billion Euro in 2005 [4]. In recognition of the burden from MSK conditions, the Swiss Federal Council’s 2016 National Strategy for the Prevention of Non-communicable Diseases [2] and its Action Plan [5] explicitly included MSK health as a priority in the context of non-communicable disease prevention and management.

The chiropractic profession in Switzerland is highly integrated into mainstream healthcare, being one of five academic health professions and having good interprofessional collaboration with other Swiss healthcare professions [6]. Healthcare for MSK conditions is covered by the mandatory Swiss health insurance and is provided by medical doctors, doctors of chiropractic medicine, and physiotherapists. Despite a promising infrastructure and the important burden due to MSK disorders, research is still regrettably rare for patients with MSK conditions seeking chiropractic care.

The Swiss federal legislation on health insurance was revised in 2019 proposing a national programme to improve the quality and safety of provided healthcare [7]. A recent national report showed insufficient availability of information and a lack of standardized quality indicators [8], which are key for successful systematic healthcare quality monitoring [9]. Specifically, patient-reported outcome measures (PROMs) are of growing importance in Switzerland and internationally [10], and as a tool for quality assurance and healthcare quality improvement [11]. PROMs are standardized tools for measuring patients' views on their health status, without interpretation of the patient's response by a clinician or anyone else [12]. By capturing patient perspectives, they are considered important tools to evaluate treatment outcomes, support shared decision-making, and enhance patient-centeredness [12]. Despite the potential benefits, implementation in routine clinical practice has some barriers, such as fear of increased work load, inappropriate training, or lack of standardized data collection [13]. Literature about the current use of PROMs among chiropractors in routine clinical practice is scarce [14], and similar settings showed only limited use of PROMs in clinical practice for patients with MSK health problems [15].

The Balgrist University Hospital, affiliated with the University of Zurich, is Switzerland’s largest specialized academic hospital focused on MSK disorders. A chiropractic medicine outpatient clinic is integrated in this interdisciplinary setting and provides chiropractic care to a broad patient population with MSK conditions. This setting presented an excellent opportunity to undertake a health services research study for the joint purposes of quality assurance and clinical epidemiological aims of investigating characteristics of MSK chiropractic care in a Swiss specialized outpatient hospital setting.

Our overall objective was to create a clinical database of chiropractic care provided at the Balgrist chiropractic medicine outpatient clinic in 2019 to advance understanding of chiropractic healthcare service for quality assurance and healthcare quality improvement. Specifically, we aimed to: (1) characterise patients seeking MSK healthcare at the Balgrist chiropractic medicine clinic, (2) describe the prevalence of MSK conditions seen at the Balgrist chiropractic medicine clinic, (3) describe characteristics of MSK care provided, and (4) assess the current quality of routine clinical healthcare data collection in the clinic.

## Methods

### Study design

We conducted an observational clinical cohort database study to describe MSK healthcare at a Swiss university hospital chiropractic medicine outpatient clinic in 2019. Our study is reported according to the Strengthening the Reporting of Observational Studies in Epidemiology (STROBE) Statement [16]. The study was reviewed and received ethical approval by the independent research ethics committee of Canton Zurich (BASEC-Nr: 2020-00361). Given the deidentified processing of these health-related data, further use in the absence of informed consent and information was granted by the Canton Zurich research ethics committee pursuant to Art. 34 of the Swiss Federal Act on Research involving Human Beings (Human Research Act, HRA). All methods followed relevant guidelines and regulations.

### Setting

The chiropractic medicine outpatient clinic is embedded in the Balgrist University Hospital—a large, academic, MSK specialized hospital—in Zurich, Switzerland. Chiropractic care in the outpatient clinic is provided by the following three groups of chiropractic clinicians and clinicians-in-training: (1) senior chiropractors (fully licensed clinicians), (2) residents undergoing their postgraduate training (academic clinicians-in-training), (3) chiropractic students (underassistants) completing a 6-month internship under supervision of experienced chiropractors in the embedded teaching clinic during the 6th year of their Masters chiropractic medicine degree program at the University of Zurich. A total of four residents see and treat patients independently and consult the attending clinician only if needed. A total of 6 students (underassistants) work and treat patients under supervision of 6 experienced senior chiropractors. The frequency of care is determined by the treating clinician.

### Source population

We prespecified our eligible study population as all patients with an initial consultation or returning initial consultation at the Balgrist chiropractic medicine clinic, from January 1, 2019, to December 31, 2019. A returning initial consultation was defined, based on hospital internal policy, as an initial consultation more than 3 months since the patient’s last visit.

### Data sources

The electronic health records of all eligible patients available through the hospital’s clinical information system (KISIM) were used to create the study database. KISIM is an integrated, comprehensive system designed to manage all hospital operations and to store information about every patient's health history. Each patient has a unique numeric identifier assigned in KISIM which allows individual patient-level identification and data linkage. Electronic imaging and reports are stored in the Picture Archiving and Communication System (PACS). Administrative data about case data and electronic invoicing of each patient is captured in the hospital billing system database (OPALE).

### Data extraction

We extracted all records for initial and follow up consultations, reports and clinical documentation notes for our study population from KISIM. Outcome data from clinical documentation notes were extracted for the study period and up to 3 months after the end of the study period (i.e., March 31, 2020). Additionally, data about other healthcare services provided at Balgrist University Hospital (i.e., physiotherapy prescriptions, corticosteroid infiltrations) were extracted for the study period and up to 3 months prior to the start of the study period (i.e., October 1, 2018). Administrative data of all internally and externally conducted imaging stored in the PACS of Balgrist University Hospital were extracted from January 1, 2018, to December 31, 2019. Information on all surgeries performed at Balgrist University Hospital was extracted for the study period and up to 5 years prior to study inception date.

Variables of interest available in KISIM were demographic characteristics, diagnoses, imaging data, conservative treatments, surgeries, and other clinical healthcare data. We include a description of all variables and their data sources in the Additional file 1. If not further specified below, the data extraction process was conducted as follows: First, data were extracted programmatically by an IT specialist from the KISIM system. Second, if programmatic data extraction was not workable or could not provide the level of information desired, then data were extracted using keyword search terms. Third, data were extracted by manual review of KISIM records or structured data elements when programmatic or keyword search approaches were not successful. For example, information about the civil status was extracted by the IT specialist from the personal data section in KISIM. If data could not be programmatically extracted from the "civil status" data field, keyword search terms such as "married" or "single" were used to find and extract the information from the patient history section of the initial visit report.

### Main variables

#### Insurance status

The insurance status of the patient was extracted from OPALE with three response options: “General” (i.e., the mandatory general health insurance coverage in Switzerland); “Semi-private”; or, “Private” (both upgraded health insurance plans with more coverages).

#### Work status

Patient's work status was classified into one of the following categories: Employed, self-employed, student/trainee, homemaker, retired, unemployed, or disability pensioner or applicant. The "employed" status was further specified according to major groups of the International Standard Classification of Occupations (ISCO-88) [17].

#### Diagnosis-related data

Diagnoses were classified according to the International Statistical Classification of Diseases and Related Health Problems 10th Revision (ICD-10) [18]. The ICD-10 code of the main diagnosis was first extracted from OPALE. If the diagnosis code was missing in OPALE, we tried to extract the diagnosis programmatically from clinical documentation notes in KISIM using keyword search terms, or by manual review of the electronic health record if the programmatic approach was unsuccessful. Up to nine additional diagnoses were extracted from the initial visit reports and converted to the most relevant and applicable ICD-10 code.

There are many distinct ICD-10 codes to describe neck or back pain. For analysis purposes, ICD-10 codes of the main diagnosis characterizing spinal pain disorders were aggregated into broader categories, e.g., neck pain, low back pain, or back pain with multiple locations. The concept for the ICD-10 codes grouping process is provided in Additional file 2. Other diagnoses not related to spinal conditions were not aggregated into broader categories as most of these referred to a specific condition (e.g., plantar fasciitis). All ICD-10 codes of the category "S" (i.e., injuries) were labelled as trauma-related diagnoses.

Duration of main diagnosis (i.e., acute, subacute or chronic) was programmatically extracted from the main diagnosis information or by using keyword search terms from the patient history of the initial consultation report. We conceptualised symptom duration information, as is commonly done in the back pain literature [19], as acute (< 4 weeks), subacute (4–12 weeks), and chronic (> 12 weeks). We extracted pain intensity at initial visit programmatically using "NRS" as a keyword search term from initial consultation reports.

#### Clinical outcomes

We extracted clinical outcomes of chiropractic care programmatically using keyword search terms for common clinical outcome measures from clinical documentation notes, progress reports, or final discharge reports. We prioritized data sources for clinical outcome extraction as follows: (1) final discharge report, (2) progress report, or (3) clinical documentation notes. The available outcome of the latest possible visit date related to the episode of care was extracted. The extracted clinical outcomes were then recoded into the following four assigned clinical outcome levels: “Worse”, “No change”, “Some improvement”, “Much improvement” (see Additional file 3: eTable 1 for details on the clinical recoding process from extracted clinical outcome to assigned clinical outcome level).

### Statistical analysis

We used descriptive statistics to summarise the data as appropriate. Exploratory subgroup comparisons of number of visits and duration of treatment period were done by main diagnosis and experience level of healthcare provider. To examine associations between patient characteristics and clinical outcomes, we used multivariable logistic regression models (complete case analysis) to estimate odds ratios (ORs) and 95% CIs, with age, gender, insurance status and work status as predictor variables. To create the binary outcome variable for logistic regression analyses, we recoded the clinical outcome levels "much improvement" and "some improvement" as "positive outcome", and "no change" and "worse" as "negative outcome". To examine associations between diagnosis characteristics and outcomes, main diagnosis, symptom duration, and related trauma were used as predictor variables of the model. Post hoc sensitivity analyses were conducted based on multiple imputation for missing outcome data, using all patient and clinical characteristics (age group, gender, insurance status, work status, diagnosis, and symptom duration), and clinical outcomes data to generate 60 imputed data sets [20, 21]. We imputed datasets with five iterations, using the multivariate imputation by chained equations algorithm in the *mice* R package [22]. All analyses were performed using R version 3.6.1 (R Foundation for Statistical Computing) [23].

## Results

Between January 1, 2019, and December 31, 2019, 1844 patients with an initial visit or return initial visit consulted for healthcare at the Balgrist chiropractic medicine polyclinic and were included in our analysis. During the study period, 1742 patients had a single initial visit, 101 had two initial visits, and one patient had three initial visits, for a total of 1947 initial visit records. The mean age of our study population was 48 ± 17 years (IQR, 35–59 years). Table 1 presents characteristics of the study population of 1844 distinct patients. Profession classifications according to the ISCO88 framework for employed work status are detailed in Additional file 3: eTable 2. The number of patients with missing data was between 0 and 1085 (58.8%) for patient characteristics (Table 1).

**Table 1:** Characteristics of 1844 patients with initial visits presenting to Balgrist University Hospital chiropractic polyclinic in 2019

Characteristic	N	%	95% CI
*Gender (n = 1844; no missing data)*			
Female	965	52.3	50.0–54.6
Male	879	47.7	45.4–50.0
*Age (years) (n = 1844; no missing data)*			
≤ 19	61	3.3	2.6–4.2
20–29	211	11.4	10.1–13.0
30–39	349	18.9	17.2–20.8
40–49	395	21.4	19.6–23.4
50–59	386	20.9	19.1–22.8
60–69	225	12.2	10.8–13.8
70–79	167	9.1	7.8–10.5
≥ 80	50	2.7	2.1–3.6
*Work status [n = 1489; missing data = 355 (19.2%)]*			
Employed	1216	81.7	79.6–83.5
Self-employed	12	0.8	0.5–1.4
Student/trainee	75	5.0	4.0–6.3
Homemaker	36	2.4	1.8–3.3
Retired	100	6.7	5.6–8.1
Unemployed	25	1.7	1.1–2.5
Disability pensioner or applicant	25	1.7	1.1–2.5
*Civil status [n = 759; missing data = 1085 (58.8%)]*			
Married	462	60.9	57.4–64.3
Single	210	27.7	24.6–31.0
Divorced	54	7.1	5.5–9.2
Widowed	16	2.1	1.3–3.4
Common law	10	1.3	0.7–2.4
Separated	7	0.9	0.4–1.9
*Insurance status [n = 1652; missing data = 192 (10.4%)]*			
General	1205	72.9	70.7–75.0
Semi-private	271	16.4	14.7–18.3
Private	176	10.7	9.3–12.2

### Characteristics of chiropractic care

Data for all initial visit records (n = 1947) related to referral and treatment are presented in Table 2. Many patient referrals to the chiropractic polyclinic—769 out of 1329 with available data; 58%—were internal referrals within Balgrist University Hospital, of which 632 (82%) were from the spine surgery division. This was followed by patient self-referrals (33%) and referrals from external general practitioners (10%). The median number of visits was 5 (IQR, 2–9 visits; range, 1–55 visits), with a median treatment episode duration of 28 days (IQR, 7–71 days; range, 0–350 days). The number of initial visit records with missing data was between 0 and 618 (31.7%) for referral and treatment characteristics (Table 2). Only 869 out of 1947 initial visit records (45%) had a recorded NRS value for pain intensity in the initial visit report, with a median pain intensity NRS of 8 (IQR, 6–9; range 0–10). Details on pain intensity reported in initial visit reports are presented in Table 2.

**Table 2:** Characteristics of 1,947 initial visit records at Balgrist University Hospital chiropractic polyclinic in 2019

Variable	N	%	95% CI
*Referral sources [n = 1329; missing data = 618 (31.7%)]*			
Internal	769	57.9	55.2–60.5
Spine surgery division	632	82.2	79.3–84.7
Other orthopaedic divisions	81	10.5	8.6–12.9
Rheumatology	22	2.9	1.9–4.3
Sports medicine	22	2.9	1.9–4.3
Others	12	1.6	0.9–2.7
External	127	9.6	8.1– 11.3
General practitioner	89	70.1	61.6–77.4
Chiropractor	15	11.8	7.3–18.6
Gynaecologist	6	4.7	2.2–9.9
Rheumatologist	5	3.9	1.7–8.9
Neurosurgeon	4	3.1	1.2–7.8
Others	12	6.3	3.2–11.9
Self-referral	433	32.6	30.1–35.1
*Treatment provider (n = 1947; no missing data)*			
Intern	907	46.6	44.4–48.8
Resident	564	29.0	27.0–31.0
Senior chiropractor	476	24.4	22.6–26.4
*Number of visits (n = 1947; no missing data)*			
1–3	739	38.0	35.8–40.1
4–7	573	29.4	27.4–31.5
8–11	304	15.6	14.1–17.3
12–15	156	8.0	6.9–9.3
16–19	88	4.5	3.7–5.5
20–29	68	3.5	2.8–4.4
30–39	17	0.9	0.5–1.4
≥ 40	2	0.1	0.0–0.4
*NRS reported in initial visit report* *(n = 869, missing data = 1078 (55.4%))*			
0	3	0.3	0.1–1.0
1	2	0.2	0.1–0.8
2	15	1.7	1.0–2.8
3	13	1.5	0.9–2.5
4	38	4.4	3.2–5.9
5	66	7.6	6.0–9.5
6	91	10.5	8.6–12.7
7	150	17.3	14.9–19.9
8	256	29.5	26.5–32.6
9	102	11.7	9.8–14.0
10	133	15.3	13.1–17.9

**Fig. 1:**
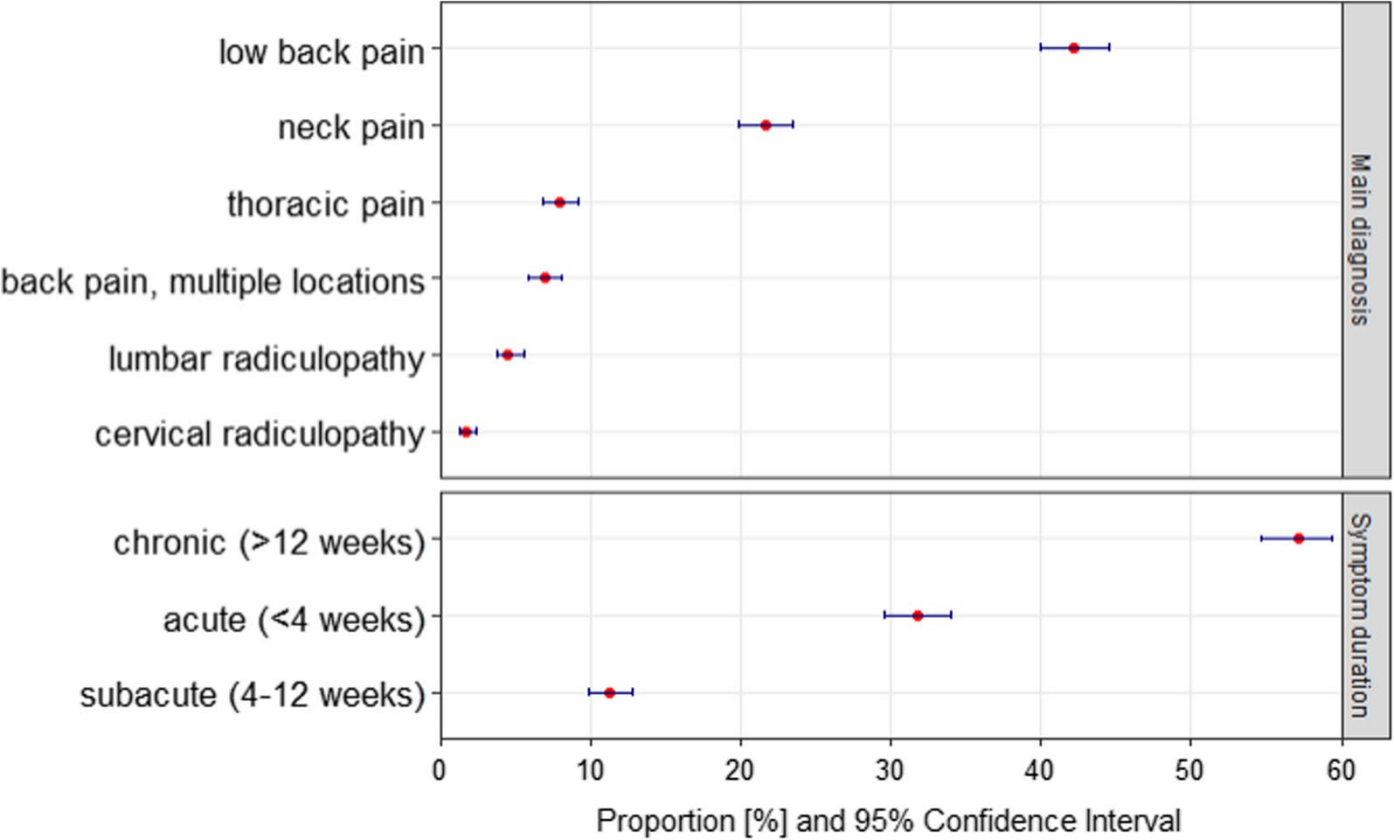
Frequency of 6 most common main diagnoses and duration of symptoms among 1947 initial visit records at Balgrist University Hospital chiropractic polyclinic in 2019. 1878 initial visit records (96.5%) with available data for diagnosis and 1770 initial visit records (90.9%) with available data for symptom duration

The frequency distribution of the 6 most common main diagnoses and duration of symptoms are detailed in Fig. 1. The most common main diagnoses among 1878 initial visit records with available data were low back pain (42%), neck pain (22%), thoracic pain (8%), and back pain with multiple locations (7%), followed by lumbar and cervical radiculopathies (5% and 2%, respectively), and other diagnoses (14%). The 10 most common other main diagnoses are presented in Additional file 3: eTable 3. Most patients in our study population (57%) presented with a chronic symptom duration, compared to 32% with acute and 11% with subacute symptoms. 6% of the diagnoses were trauma or injury related. The number of initial visit records with missing data was 69 (3.5%) for main diagnosis and 177 (9.1%) for duration of symptoms (Fig. 1).

The number of treatment visits and duration of treatment episode according to the 6 most common main diagnoses and treatment provider groups are presented in Table 3. The most intensive chiropractic care was provided to patients with cervical radiculopathy with an average of 10 treatment visits within 84 days, followed by lumbar radiculopathy with 9 visits within 70 days. There was minor difference in the number of visits and treatment durations between the three types of providers.

**Table 3:** Average number of visits and treatment episode durations for the most common diagnoses and treatment provider types from 1947 initial visit records at Balgrist University Hospital chiropractic polyclinic in 2019

	Number of visits (mean ± SD)	Treatment duration [days] (mean ± SD)
*6 most common main diagnoses [n = 1878, missing data = 69 (3.5%)]*		
Cervical radiculopathy	10 ± 8	84 ± 87
Lumbar radiculopathy	9 ± 8	70 ± 75
Back pain, multiple locations	8 ± 7	64 ± 75
Neck pain	7 ± 6	54 ± 64
Low back pain	7 ± 6	50 ± 61
Thoracic pain	5 ± 5	41 ± 60
*Treatment provider (n = 1947, no missing data)*		
Intern	7 ± 6	51 ± 63
Resident	6 ± 6	48 ± 60
Senior chiropractor	7 ± 7	59 ± 71

### Patient-reported clinical outcomes

Among 1947 initial visit records in our study population, 986 (50.6%) had no recorded clinical outcome that was extractable. Among 961 initial visit records with an extractable clinical outcome, 46% (95% CI 43–50%) reported “much improvement”, 41% (38–45%) “some improvement”, 11% (10–14%) “no change”, and 0.8% (0.4–1.6%) “worse”. Details on clinical outcome sources are reported in Additional file 3: eTable 4, and a comparison of participants with or without an extractable clinical outcome is presented in Additional file 3: eTable 5.

### Associations between initial visit characteristics and clinical outcome

Our logistic regression analysis (n = 764 complete cases) examining associations between patient characteristics and clinical outcome found an association between younger age (age group 20–29 years versus referent group 40–49 years) and positive clinical outcome (OR 2.2, 95% CI 1.0–5.5) (Additional file 3: eTable 6). Our complete case analysis was compatible with no associations between clinical outcome and gender, work status, or insurance status. The multiple imputation sensitivity analysis based on the obtained-plus-imputed characteristics and outcome data showed similar results as the complete case analysis (Additional file 3: eTable 6).

Due to the vast number of different ICD-codes, only the 5 most frequent diagnoses were evaluated in more detail. The logistic regression model (n = 760 complete cases) examining associations between diagnosis characteristics and clinical outcome suggested that an acute symptom duration (< 4 weeks) was associated with positive clinical outcome (OR 4.0, 95% CI 1.2–14.0) compared to the referent subacute symptom duration (Additional file 3: eTable 7). Our data were compatible with no associations for clinical outcome by main diagnosis or trauma-related clinical presentations. Our multiple imputation sensitivity analysis showed very similar results (Additional file 3: eTable 7).

### Other healthcare services utilization

Among our study population of 1844 distinct patients, 998 (54%, 95% CI 52–56%) received at least one physiotherapy prescription, and 343 (19%, 17–20%) at least one work disability certificate. 726 (39%, 37–42%) of the study population patients received at least one X-ray and 481 (26%, 24–28%) received at least one MRI of the spine during the treatment episode at the chiropractic medicine clinic or up to one month prior to their initial visit. Of all spinal X-ray images taken (n = 904), 47% (43–50%) were of the lumbar spine, 35% (32–38%) of the cervical spine, 13% (11–16%) of the whole spine, and 5% (4–7%) of the thoracic spine. Of all spinal MRI services taken (n = 553), the most common MRI was for the lumbar spine (66%, 62–70%), followed by the cervical spine (24%, 20–27%). 50 (3%, 2–4%) of the patients received an imaging-guided corticosteroid injection of the spine during their treatment episode ordered by the chiropractor. There were 49 patients (3%, 95% CI 2–4%) with a history of spine surgery 12 months prior to their initial visit, and 13 patients (0.4%, 0.2–0.8%) who underwent spine surgery within 12 months after their initial visit at the chiropractic medicine polyclinic.

## Discussion

Our health services research study provides an initial understanding of patient characteristics and MSK clinical care delivered in a Swiss university based MSK specialized hospital chiropractic outpatient setting. We found that only 49% of the initial visits records in 2019 had a PROM that was able to be extracted from routine clinical practice documentation available in the hospital clinical information system (KISIM). Our analysis found not only a high amount of missing PROM data, but also some high amounts of missing sociodemographic and clinical information (e.g., 59% for civil status, 19% for profession, and 55% for initial visit pain intensity NRS).

The demographics and presenting main diagnoses for our study population were similar to other studies. The slightly higher percentage of women (52%) seeking chiropractic care has been described previously, whereas our patient population with the most common age group of 40–49 years (21.4%), followed by 50–59 years (20.9%), seems to be older compared to other chiropractic settings [24]. The most common main diagnosis of low back pain, followed by neck pain, is consistent internationally [24] and in other chiropractic teaching clinics [25–27]. Patients averaged 7 chiropractic visits during their episode of care, with 83% having 11 visits or less. While 95% of chiropractic teaching institutions report the routine use of PROMs for low back pain patients [28], evidence about the current use of PROMs among chiropractors in routine clinical practices is limited [14]. One study reported that almost 30% of a chiropractor population in Australia don't routinely assess PROMs in clinical practice for low back pain patients [29]. Other primary care MSK settings have reported that 46% [30] and 60% [31] of physiotherapists use PROMs.

As in many healthcare settings, most of our data were stored in text form in reports and clinical documentation notes. In most healthcare settings, these electronic data sources are typically unstructured, heterogeneous, and incomplete [32]. Other barriers for the routine use of PROMs are the additional work load associated with data collection and lack of clear guidelines on the data collection process (e.g. frequency, timing, and responsible personnel for data collection) [13]. The high missingness of sociodemographic information in our study is likely due to patients often not providing complete information on nonmandatory disclosures on the personal data sheet required for the initial visit and/or the data not being adequately transcribed into the electronic health record system by administrative staff [32].

PROMs are important standardized tools to measure the effectiveness of patient-centred care [12], and evidence of their value in improving individual care [11] and healthcare quality [33, 34] is increasing. Internationally, there is growing interest in the role of PROMs in facilitating quality improvement initiatives and focusing patient-centred and patient-relevant healthcare outcomes [10]. To achieve best care, reducing inequities in provided healthcare is crucial [35]. The linkage of patient-level outcomes with sociodemographic data is key for health equity monitoring to provide equitable access to high quality care [36]. One of the three recommendations of the World Health Organization's Commission on the Social Determinants of Health in 2008 was to "measure and understand the problem and assess the results of action", stating hereby the need for routine data collection and monitoring systems [37].

### Strengths and limitations

A strength of our study was the inclusion of a relatively large unselected MSK patient population presenting to the Balgrist chiropractic polyclinic during 2019, so that it captures the heterogeneous sample of patients seeking MSK healthcare in a Swiss university hospital chiropractic medicine outpatient clinic. Our focus was descriptive in nature for the purpose of quality assurance and future healthcare quality improvement. Collection and analysis of real-world data facilitates integration of research findings into routine clinical practice.

The external validity of our study is limited by the single-centre approach, and our findings may not be generalizable to other chiropractic outpatient settings. As data collection and data entry in retrospective cohort studies are not planned in advance, our data were limited by the information available and extractable from the electronic clinical information system. Missing data limited our ability to fully describe all characteristics of our study population and all characteristics of the MSK care provided. Our chosen cutpoint of > 80% improvement for the assigned outcome level "much improvement" may have underestimated the number of persons with much improvement as an outcome, while our broad specification of "some improvement" as > 0% to < 80% improvement may have overestimated those with some improvement. Since the available outcome of the latest possible visit date related to the episode of care was extracted, the intensity of chiropractic care varied.

### Implications

Our study provides insights on routinely collected clinical data about healthcare services and PROMs at a university hospital chiropractic medicine outpatient clinic in 2019. By assessing the current data structure, quality, and accessibility, we were able to identify data collection quality and performance gaps. The following overall aims and targets were set for future health quality improvement initiatives at the Balgrist chiropractic medicine clinic: (1) To improve the quality and structure of routine clinical documentation practices, (2) To better integrate routine electronic PROM collection into routine clinical practice, and (3) To implement data quality evaluation and monitoring processes for quality assurance and chiropractic healthcare quality improvement.

Integrating a new routine into daily clinical practice takes time and effort, and there are several possible challenges and barriers to overcome such as PROMs perceived as too time consuming and not user friendly, and lack of training and knowledge about their use [29]. To ensure successful implementation, we aim to address the following facilitators: [38] (1) Incorporation of PROMs into existing workflows, (2) utilisation of simple administration systems and basic electronic forms, and (3) clear guidelines of PROMs use and sufficient training of clinicians.

Specifically, we are developing a new standardized tool for routine clinical data collection, integrated into the hospital's patient information system. The tool is designed to capture clinical notes (e.g., type and location of treatment) and brief, feasible PROMs (e.g., PGIC and NRS) in a structured format. This may facilitate the collection of higher quality clinical information that could be easily extracted for meaningful use and analysis. Furthermore, we started to develop processes to assess and monitor the data quality of initial visit reports. Quality assurance reports have been implemented within the chiropractic medicine clinic on a quarterly basis to measure the amount of missing data, for example, for ICD diagnosis codes, duration of symptoms on the initial visit report, and missing PROMs in the clinical notes. Such audit and feedback processes have been shown to be useful to improve healthcare professionals’ performance [39].

## Conclusion

Our health services study provides an initial understanding of the patient characteristics and healthcare delivered in a Swiss academic hospital chiropractic outpatient setting and areas for improved clinical data quality assurance. A more concerted effort to systematically collect patient reported outcome measures would be a worthwhile healthcare quality improvement initiative.

## Supplementary Information

**Additional file 1:** Data dictionary.

**Additional file 2:** Concept for ICD-10 codes grouping process.

**Additional file 3:** Supplementary tables.

## Data Availability

The datasets used and/or analysed during the current study are available from the corresponding author on reasonable request.

## Abbreviations

ICD-10: International Statistical Classification of Diseases and Related Health Problems 10th Revision
ISCO: International Standard Classification of Occupations
MSK: Musculoskeletal
OPALE: Balgrist hospital billing system database
PACS: Picture Archiving and Communication System
PGIC: Patient global impression of change
PROM: Patient-reported outcome measure

## References

[1] James SL, Abate D, Abate KH (2018). Global, regional, and national incidence, prevalence, and years lived with disability for 354 diseases and injuries for 195 countries and territories, 1990–2017: a systematic analysis for the Global Burden of Disease Study 2017. The Lancet.

[2] Federal Office of Public Health (FOPH), Swiss Conference of the Cantonal Ministers of Public Health (CMPH). The challenge of non-communicable diseases: National Strategy on the Prevention of Non-Communicable Diseases 2017–2024 (NCD Strategy), short version [Internet]. Bern: Federal Office of Public Health (FOPH); 2016 [cited 2019 Nov 12]. https://www.bag.admin.ch/dam/bag/en/dokumente/nat-gesundheitsstrategien/ncd-strategie/ncd-kurzversion.pdf.download.pdf/ncd-kurzversion.pdf.

[3] Wieser S, Tomonaga Y, Riguzzi M, et al. Die Kosten der nichtübertragbaren Krankheiten in der Schweiz: Schlussbericht. 2014 [cited 2021 Oct 12]. http://www.bag.admin.ch/themen/medizin/00683/.

[4] Wieser S, Horisberger B, Schmidhauser S (2011). Cost of low back pain in Switzerland in 2005. Eur J Health Econ.

[5] Federal Office of Public Health (FOPH), Swiss Conference of the Cantonal Ministers of Public Health (CMPH), Health Promotion Switzerland (HPS). Overview of action plan accompanying the National Strategy on the Prevention of Non-Communicable Diseases (NCD strategy) 2017–2024 [Internet]. Bern: Federal Office of Public Health; 2016 [cited 2019 Nov 12]. https://www.bag.admin.ch/dam/bag/en/dokumente/nat-gesundheitsstrategien/ncd-strategie/ncd-massnahmenplan.pdf.download.pdf/ncd-massnahmenplan.pdf.

[6] Humphreys BK, Peterson CK, Muehlemann D, Haueter P (2010). Are Swiss chiropractors different than other chiropractors? Results of the job analysis survey 2009. J Manip Physiol Ther.

[7] Bundesgesetz über die Krankenversicherung (KVG) (Stärkung von Qualität und Wirtschaftlichkeit). Änderung vom 21. Juni 2019 [Internet]. 2019 [cited 2021 Mar 23]. https://www.fedlex.admin.ch/eli/fga/2019/1584/de.

[8] Vincent C, Staines A. Enhancing the quality and safety of Swiss healthcare. A national report. [Internet]. 2019 [cited 2021 Jul 29]. https://www.bag.admin.ch/bag/de/home/versicherungen/krankenversicherung/qualitaetsentwicklung-schweiz.html.

[9] Elixhauser A, Pancholi M, Clancy CM (2005). Using the AHRQ quality indicators to improve health care quality. Jt Comm J Qual Patient Saf.

[10] Van Der Wees PJ, Nijhuis-Van Der Sanden MWG, Ayanian JZ, Black N, Westert GP, Schneider EC (2014). Integrating the use of patient-reported outcomes for both clinical practice and performance measurement: views of experts from 3 countries. Milbank Q.

[11] Greenhalgh J, Dalkin S, Gibbons E (2018). How do aggregated patient-reported outcome measures data stimulate health care improvement? A realist synthesis. J Health Serv Res Policy.

[12] Ahmed S, Berzon RA, Revicki DA (2012). The use of patient-reported outcomes (PRO) within comparative effectiveness research: implications for clinical practice and health care policy. Med Care.

[13] Boyce MB, Browne JP, Greenhalgh J (2014). The experiences of professionals with using information from patient-reported outcome measures to improve the quality of healthcare: a systematic review of qualitative research. BMJ Qual Saf.

[14] Clohesy NC, Schneiders AG, Eaton S (2018). Utilization of low back pain patient reported outcome measures within chiropractic literature: a descriptive review. J Manip Physiol Ther.

[15] Meerhoff GA, Dulmen SA van, Maas MJM, Bakker-Jacobs A, Sanden MWGN-V der, Wees PJ van der. Exploring the perspective of patients with musculoskeletal health problems in primary care on the use of patient-reported outcome measures to stimulate quality improvement in physiotherapist practice; a qualitative study. Physiother Theory Pract 2019;37(9):1–12. 10.1080/09593985.2019.1678205 31635516

[16] von Elm E, Altman DG, Egger M (2007). The strengthening the reporting of observational studies in epidemiology (STROBE) statement: guidelines for reporting observational studies. Lancet Lond Engl.

[17] Bureau of Statistics, work unit of the Policy Integration Department [Internet]. [cited 2021 Feb 4]. https://www.ilo.org/public/english/bureau/stat/isco/isco88/index.htm.

[18] Office of the Secretary, HHS. Administrative simplification: change to the compliance date for the international classification of diseases, 10th revision (icd-10-cm and icd-10-pcs) medical data code sets. final rule. Fed Regist 2014;79(149):45128–34. 25122944

[19] Qaseem A, Wilt TJ, McLean RM (2017). Noninvasive treatments for acute, subacute, and chronic low back pain: a clinical practice guideline from the american college of physicians. Ann Intern Med.

[20] White IR, Royston P, Wood AM (2011). Multiple imputation using chained equations: issues and guidance for practice. Stat Med.

[21] Sterne JAC, White IR, Carlin JB (2009). Multiple imputation for missing data in epidemiological and clinical research: potential and pitfalls. The BMJ.

[22] van Buuren S, Groothuis-Oudshoorn K (2011). Mice: multivariate imputation by chained equations in R. J Stat Softw.

[23] R: The R Project for Statistical Computing [Internet]. [cited 2021 Feb 3]. https://www.r-project.org/.

[24] Beliveau PJH, Wong JJ, Sutton DA (2017). The chiropractic profession: a scoping review of utilization rates, reasons for seeking care, patient profiles, and care provided. Chiropr Man Ther.

[25] Stevens G, Campeanu M, Sorrento AT, Ryu J, Burke J (2016). Retrospective demographic analysis of patients seeking care at a free university chiropractic clinic. J Chiropr Med.

[26] Martinez DA, Rupert RL, Ndetan HT (2009). A demographic and epidemiological study of a Mexican chiropractic college public clinic. Chiropr Osteopat.

[27] Ismail F, Booysen N, Yelverton C, Peterson C (2020). Characteristics of chiropractic patients treated at the University of Johannesburg chiropractic student clinic and relevance to the educational process. J Chiropr Educ.

[28] Cooper JC, Gliedt JA, Pohlman KA (2020). A descriptive analysis of clinical application of patient-reported outcome measures and screening tools for low back pain patients in US chiropractic teaching institutions. J Chiropr Educ.

[29] Clohesy N, Schneiders A (2018). A preliminary investigation examining patient reported outcome measures for low back pain and utilisation amongst chiropractors in Australia: facilitators and barriers to clinical implementation. Chiropr Man Ther.

[30] Brinkman M, Barten D, Pisters M, Verheij R (2019). Current use of PROMs and factors associated with their use in patients with nonspecific low back pain. Learn Health Syst.

[31] van Dulmen SA, van der Wees PJ, Bart Staal J, Braspenning JCC, Nijhuis-van der Sanden MWG. Patient reported outcome measures (PROMs) for goalsetting and outcome measurement in primary care physiotherapy, an explorative field study. Physiotherapy 2017;103(1):66–72. 10.1016/j.physio.2016.01.001 27033783

[32] Hripcsak G, Albers DJ (2013). Next-generation phenotyping of electronic health records. J Am Med Inform Assoc JAMIA.

[33] Black N (2013). Patient reported outcome measures could help transform healthcare. BMJ.

[34] Zhang R, Burgess ER, Reddy MC (2019). Provider perspectives on the integration of patient-reported outcomes in an electronic health record. JAMIA Open.

[35] Mayberry RM, Nicewander DA, Qin H, Ballard DJ (2006). Improving quality and reducing inequities: a challenge in achieving best care. Proc Bayl Univ Med Cent.

[36] Kirst M, Shankardass K, Bomze S, Lofters A, Quiñonez C (2013). Sociodemographic data collection for health equity measurement: a mixed methods study examining public opinions. Int J Equity Health.

[37] Marmot M, Friel S, Bell R, Houweling TA, Taylor S (2008). Closing the gap in a generation: health equity through action on the social determinants of health. The Lancet.

[38] Foster A, Croot L, Brazier J, Harris J, O’Cathain A (2018). The facilitators and barriers to implementing patient reported outcome measures in organisations delivering health related services: a systematic review of reviews. J Patient-Rep Outcomes.

[39] Ivers N, Jamtvedt G, Flottorp S (2012). Audit and feedback: effects on professional practice and healthcare outcomes. Cochrane Database Syst Rev.

