# Supplementary material for: Musculoskeletal healthcare at a Swiss university hospital chiropractic medicine outpatient clinic in 2019: a health services research study
Source: Chiropr Man Therap. 2022 Feb 11;30:7. doi: 10.1186/s12998-022-00417-5 (PMC8832828; doi:10.1186/s12998-022-00417-5)
Supplement: Supplementary file 3 — Additional file 3: Supplementary tables. [file 12998_2022_417_MOESM3_ESM.docx]

## Additional File 3

## eTable 1. Recoding of extracted clinical outcome to assigned outcome level

| **Clinical outcome** | **Assigned outcome level** |
| --- | --- |
| **Patient global impression of change (PGIC)** | |
| 1 (very much improved) | Much improvement |
| 2 (much improved), 3 (minimally improved) | Some improvement |
| 4 (no change) | No change |
| 5 (minimally worse), 6 (much worse), 7 (very much worse) | Worse |
| **Percentage improvement** | |
| ≥80% improvement | Much improvement |
| >0% to <80% improvement | Some improvement |
| 0% | No change |
| Negative % | Worse |
| **Keyword terms in German [English]** |  |
| Beschwerdefreiheit, beschwerdefrei [Pain free] | Much improvement |
| Keine Schmerzen [No pain] | Much improvement |
| Sehr gut [Very good] | Much improvement |
| Deutliche Regredienz [Significant resolution] | Some improvement |
| Deutliche Beschwerdelinderung [Significant pain relief] | Some improvement |
| Gut [Good] | Some improvement |
| Keine Verbesserung [No improvement] | No change |
| Keine Veränderung, unverändert [No change] | No change |
| Vermehrte Schmerzen [More pain] | Worse |
| Verschlimmerung [Aggravation] | Worse |

## eTable 2. ISCO88 classification of profession among 1216 initial visit patients reporting employed work status

| **Variable** | **N** | **%** | **95% CI** |
| --- | --- | --- | --- |
| 1 Legislators, senior officials and managers | 72 | 5.9 | 4.7 – 7.4 |
| 2 Professionals | 278 | 22.9 | 20.6 – 25.3 |
| 3 Technicians and associate professionals | 275 | 22.6 | 20.4 – 25.1 |
| 4 Clerks | 210 | 17.3 | 15.2 – 19.5 |
| 5 Service workers and shop and market sales workers | 107 | 8.8 | 7.3 – 10.5 |
| 6 Skilled agricultural and fishery workers | 9 | 0.7 | 0.4 – 1.4 |
| 7 Craft and related trade workers | 122 | 10.0 | 8.5 – 11.8 |
| 8 Plant and machine operators and assemblers | 45 | 3.7 | 2.8 – 4.9 |
| 9 Elementary occupations | 98 | 8.1 | 6.7 – 9.7 |
| 0 Armed forces | 0 | 0 | - |

## eTable 3. 10 most common initial visit other main diagnoses

| **Variable** | **N** | **%** | **95% CI** |
| --- | --- | --- | --- |
| Muscle disorder | 24 | 1.3 | 0.9 – 1.9 |
| Cervicogenic headache | 19 | 1.0 | 0.6 – 1.6 |
| Plantar fasciitis | 15 | 0.8 | 0.5 – 1.3 |
| Adhesive capsulitis of shoulder | 10 | 0.5 | 0.3 – 1.0 |
| Achilles tendinitis | 10 | 0.5 | 0.3 – 1.0 |
| Dizziness | 10 | 0.5 | 0.3 – 1.0 |
| Sciatica | 10 | 0.5 | 0.3 – 1.0 |
| Calcaneal spur | 5 | 0.3 | 0.1 – 0.6 |
| Sprain and strain of shoulder joint | 5 | 0.3 | 0.1 – 0.6 |
| Joint pain, multiple locations | 4 | 0.2 | 0.1 – 0.5 |
| All other diagnoses | 173 | 9.2 | 8.0 – 10.6 |

**eTable 4. Sources of clinical outcomes among 961 initial visit records with an extractable clinical outcome in the electronic clinical information system***

| **Clinical outcome source** | **N** | **%** |
| --- | --- | --- |
| Keyword terms (see eTable 1 for details) | 585 | 60.9 |
| Progress report | 116 | 19.8 |
| Final report | 469 | 80.2 |
| % improvement | 336 | 35.0 |
| Clinical documentation notes | 276 | 82.1 |
| Progress report | 26 | 7.7 |
| Final report | 34 | 10.1 |
| Patient global impression of change (PGIC) | 40 | 4.2 |
| Clinical documentation notes | 40 | 100 |
| * In 986 initial visit records (50.6%) a clinical outcome was not able to be extracted from the electronic clinical information system. | | |

**eTable 5. Comparison of characteristics among 961 initial visit records with an extractable clinical outcome and 986 initial visit records without an extractable outcome**

|  | **Patients with an extractable outcome**  **(N=961)** | | **Patients without an extractable outcome**  **(N=986)** | |
| --- | --- | --- | --- | --- |
| **Characteristic** | **N** | **%** | **N** | **%** |
| Gender | | | | |
| Female | 506 | 52.7 | 524 | 53.1 |
| Male | 455 | 47.3 | 462 | 46.9 |
| Age (years) | | | | |
| ≤ 19 | 29 | 3.0 | 36 | 3.7 |
| 20-29 | 118 | 12.3 | 104 | 10.5 |
| 30-39 | 170 | 17.7 | 192 | 19.5 |
| 40-49 | 225 | 23.4 | 191 | 19.4 |
| 50-59 | 189 | 19.7 | 221 | 22.4 |
| 60-69 | 114 | 11.9 | 131 | 13.3 |
| 70-79 | 84 | 8.7 | 92 | 9.3 |
| ≥ 80 | 32 | 3.3 | 19 | 1.9 |
| Work status | | | | |
| Employed | 648 | 67.4 | 633 | 64.2 |
| Self-employed | 3 | 0.3 | 10 | 1.0 |
| Student/trainee | 40 | 4.2 | 38 | 3.9 |
| Homemaker | 28 | 2.9 | 10 | 1.0 |
| Retired | 67 | 7.0 | 37 | 3.8 |
| Unemployed | 17 | 1.8 | 10 | 1.0 |
| Disability pensioner or applicant | 17 | 1.8 | 10 | 1.0 |
| NA | 516 | 53.7 | 632 | 64.1 |
| Insurance status | | | | |
| General | 699 | 72.7 | 570 | 57.8 |
| Semi private | 120 | 12.5 | 170 | 17.2 |
| Private | 70 | 7.3 | 117 | 11.9 |
| NA | 72 | 7.5 | 129 | 13.1 |
| Treatment provider |  |  |  |  |
| Intern | 598 | 62.2 | 309 | 31.3 |
| Resident | 242 | 25.2 | 322 | 32.7 |
| Senior chiropractor | 121 | 12.6 | 355 | 36.0 |

**eTable 6. Associations between initial visit characteristics and positive clinical outcome for complete case (N=764) and multiple imputation analyses (N=1947)**

|  | **Complete cases**  **(N=764)** | **Multiple imputation**  **(N=1947)** |
| --- | --- | --- |
| **Characteristic** | **OR (95% CI)** | **OR (95% CI)** |
| Gender | | |
| Male | Referent | Referent |
| Female | 0.9 (0.6 to 1.4) | 1.0 (0.7 to 1.5) |
| Age group |  |  |
| 40-49 years | Referent | Referent |
| ≤19 years | 2.1 (0.5 to 15.0) | 2.2 (0.5 to 10.9) |
| 20-29 years | 2.2 (1.0 to 5.5) | 2.2 (1.0 to 5.1) |
| 30-39 years | 1.3 (0.7 to 2.6) | 1.4 (0.7 to 2.5) |
| 50-59 years | 0.8 (0.5 to 1.5) | 1.0 (0.6 to 1.8) |
| 60-69 years | 2.0 (0.8 to 5.3) | 2.0 (0.9 to 4.5) |
| 70-79 years | 1.1 (0.3 to 5.3) | 1.2 (0.4 to 3.5) |
| ≥80 years | 1.0 (0.2 to 7.9) | 0.6 (0.2 to 2.3) |
| Insurance status |  |  |
| General | Referent | Referent |
| Semi-private | 1.7 (0.8 to 3.9) | 2.0 (1.1 to 3.7) |
| Private | 2.1 (0.8 to 7.5) | 2.0 (0.8 to 4.7) |
| Work status |  |  |
| Disability pensioner/applicant | Referent | Referent |
| Employed | 1.1 (0.3 to 3.7) | 1.1 (0.3 to 4.2) |
| Homemaker | 1.0 (0.2 to 5.3) | 0.8 (0.2 to 3.9) |
| Retired | 1.7 (0.2 to 10.4) | 1.5 (0.3 to 8.7) |
| Self-employed | 0.1 (0 to 2.4) | 0.3 (0 to 5.0) |
| Student/trainee | 1.2 (0.2 to 8.7) | 1.3 (0.2 to 8.3) |
| Unemployed | 0.4 (0.1 to 2.4) | 0.4 (0.1 to 2.4) |

**eTable 7. Associations between diagnosis related characteristics and positive clinical outcome for complete case (N=760) and multiple imputation analyses (N=1561)**

|  | **Complete cases**  **(N=760)** | **Multiple imputation**  **(N=1561)** |
| --- | --- | --- |
| **Characteristic** | **OR (95% CI)** | **OR (95% CI)** |
| Symptom duration | | |
| Subacute | Referent | Referent |
| Acute | 4.0 (1.2 to 14.0) | 4.0 (1.2 to 13.1) |
| Chronic | 0.5 (0.2 to 1.1) | 0.5 (0.2 to 1.1) |
| Most common diagnoses |  |  |
| Low back pain | Referent | Referent |
| Back pain, multiple locations | 0.5 (0.2 to 1.1) | 0.5 (0.2 to 1.0) |
| Lumbar radiculopathy | 0.9 (0.4 to 2.4) | 0.9 (0.3 to 2.2) |
| Neck pain | 1.3 (0.7 to 2.5) | 1.3 (0.7 to 2.4) |
| Thoracic pain | 0.7 (0.3 to 1.6) | 0.7 (0.3 to 1.5) |
| Trauma related presentation |  |  |
| No | Referent | Referent |
| Yes | 0.9 (0.4 to 2.2) | 0.9 (0.4 to 2.1) |
